# Resistance to β-lactams in Bacteria Isolated from Different Types of Portuguese Cheese

**DOI:** 10.3390/ijms10041538

**Published:** 2009-04-07

**Authors:** Paula Amador, Ruben Fernandes, Cristina Prudêncio, Luísa Brito

**Affiliations:** 1 Laboratório de Microbiologia, CBAA/DBEB, Instituto Superior de Agronomia, Technical University of Lisbon, Tapada da Ajuda 1349-017 Lisbon, Portugal; E-Mail: paula_amador@esac.pt (P.A.); 2 Ciências Químicas e das Biomoléculas, Escola Superior de Tecnologia de Saúde do Porto, Instituto Politécnico do Porto, Praça do Coronel Pacheco, No. 15, 4050-453 Porto, Portugal; E-Mails: rpf@estsp.ipp.pt (R.F.); cps@estsp.ipp.pt (C.P.); 3 Ciências Exactas e do Ambiente, Sector de Biologia e Ecologia, Escola Superior Agrária, Instituto Politécnico de Coimbra, Bencanta, 3040-316 Coimbra, Portugal

**Keywords:** Ready-to-eat (RTE) food, cheese, minimal inhibitory concentration (MIC), β-lactam-resistant Enterobacteriaceae, multiplex-PCR, *bla*_TEM_ gene

## Abstract

The purpose of this study was to investigate the presence of β-lactam-resistant bacteria in six different types of Portuguese cheese. The numbers of ampicillin resistant (AMP^r^) bacteria varied from 4.7 × 10^2^ to 1.5 × 10^7^ CFU/g. Within 172 randomly selected β-lactam-resistant bacteria, 44 resistant phenotypes were found and 31.4% were multidrug resistant. The majority (85%) of the isolates identified belonged to the Enterobacteriaceae family. The presence of the *bla*_TEM_ gene was detected in 80.9% of the tested isolates. The results suggest that without thermal processing of the milk and good hygienic practices, cheese may act as a vehicle of transfer of β-lactam-resistant bacteria to the gastrointestinal tract of consumers.

## 1. Introduction

Antimicrobials are natural or synthetic drugs which inhibit or kill bacteria. This ability makes them unique for the control of deadly infectious diseases caused by a large variety of pathogenic bacteria [1]. Acquired antimicrobial resistance is particularly problematic and can limit treatment options, contributing to more pain and increased deaths. In humans, most of the rising antimicrobial resistance results from the overuse and misuse of antimicrobials by patients and health personnel. The widespread use of antimicrobials outside human medicine is another serious concern. In fact, antimicrobials have also been used increasingly in plant biotechnology and in animals, for the treatment of bacterial disease or as growth-enhancing compounds in intense husbandry [2]. Antimicrobials-resistant bacteria also occur in some retail meats and poultry and in diverse other types of food, including ready-to-eat (RTE) [3].

Among the newly-emerging resistant bacteria transmitted to humans, mainly via meat and other food of animal origin are the members of the family Enterobacteriaceae [3]. This family comprises a large group of Gram-negative, non-spore-forming, facultative anaerobic bacteria. Enterobacteriaceae are often associated with poor hygiene practices in the manipulation of foods of animal origin, but they may themselves represent the natural flora of the food. Milk and dairy products contain Enterobacteriaceae at variable levels. In some cheese-making processes these bacteria can contribute to flavour and texture, but their presence, particularly at high levels, may be less desirable owing to their ability to cause spoilage [4].

Different antimicrobial resistance mechanisms have been described [5–7]. One of them is the presence of hydrolytic enzymes, such as the β-lactamases that cleave β-lactam antibiotics. Rates of bacterial resistance to antimicrobial agents are increasing worldwide and the production of β-lactamases is the most common mechanism of bacterial resistance [8,9]. Different types of β-lactamases have been reported in different genera of Gram negative bacillary bacteria, with a high prevalence in members of the family Enterobacteriaceae. The two types of β-lactamases that are associated to most of the increasing multidrug resistance are the class C enzymes and the extended-spectrum class A enzymes, namely the chromosomal-encoded AmpC β-lactamases and the plasmid encoded extended-spectrum β-lactamases (ESBL) [10]. AmpC β-lactamases are expressed constitutively at very low levels and generally do not contribute to β-lactam resistance. However, in some Enterobacteriaceae (*Serratia marcescens*, *Citrobacter freundii*, *Morganella morganii*, *Providencia stuartii* and especially *Enterobacter cloacae*) AmpC β-lactamases can be induced in the presence of a β-lactam or as a result of regulatory gene mutations [11]. Nevertheless, other Enterobacteriaceae such as *Escherichia coli*, *Klebsiella pneumoniae*, have been described as harbouring plasmid AmpC β-lactamases [12]. Mesa *et al*. (2006) [13], reported a prevalence of 31.1% of resistant Enterobacteriaceae among food outbreaks with 4.4% to 66.6% of carriers within each outbreak. These data reinforce the hypothesis that ESBL producing Enterobacteriaceae could be transmitted to humans by the food supply, particularly by the consumption of RTE food that are eaten without being cooked, such as cheese. Nevertheless, resistance to β-lactams has also been reported in non-Enterobacteriaceae bacteria such as Pseudomonales [14]. The aim of this study was an investigation of the presence of antimicrobial resistance to broad spectrum β-lactams within bacteria isolated from different types of Portuguese cheese.

## 2. Experimental Section

### 2.1. Sampling

Twenty cheese samples, corresponding to six different types of Portuguese soft and semi-soft ripened cheeses were used. Table 1 shows the origin and the occurrence of thermal processing of the milk. The cheeses were random collected in 2007, at retail establishments in different regions, from the North to the South of Portugal. From each cheese, an aliquot of 25 g (which included approximately equal amounts of material from the inner and outer parts of the cheese) was removed and blended for 2 min, in a Stomacher^®^ (Barcelona, Spain), with 225 mL of buffered peptone water (BPW) (pH 7.0) supplemented with 50 μg/mL cycloheximidine (Sigma, Saint Louis, USA).

### 2.2. *Enumeration of ampicillin* resistant bacteria

The enumeration of AMP^r^ bacteria was performed on five cheese samples randomly selected from five types of cheese (A, B, C, D and F). Portions of 0.1 mL of the homogenised or of the appropriately diluted cheese samples in BPW containing 50 μg/mL cycloheximidine, obtained as described above, were immediately surface plated onto three replicate plates of VRBG (Violet Red Bile Glucose) agar (Oxoid, Hampshire, England) supplemented with 20 μg/mL of sodium ampicillin (AppliChem, Damstadt, Germany). Plates were incubated aerobically at 37 ºC for 38 h before counting the colonies. Although this medium is used to select for Enterobacteriaceae, all the AMP^r^ isolates were counted.

### 2.3. *Detection of ampicillin* resistant bacteria

In order to recover all the resistance phenotypes present in the cheeses, even stressed ones due to milk and/or cheese processing, after sampling each one of the twenty homogenised cheese samples were overnight incubated at 37 ºC, before surface plating on VRBG and on SS (*Salmonella* & *Shigella*) agar (Oxoid, Hampshire, England). Both media were supplemented with 20 μg/mL of sodium ampicillin (AppliChem, Damstadt, Germany). Plates were incubated aerobically at 37 ºC for 24 to 48 h. Different morphological types of AMP^r^ colonies were selected and replicated five times for phenotypic confirmation. Strains *Escherichia coli* XL1-Blue and *Escherichia coli* XL1-Blue transformed with plasmid pUC18 (which contains the TEM-1 β-lactamase gene) were used as negative and positive controls, respectively for AMP^r^ phenotype identification.

### 2.4. Nitrocefin test and antimicrobial susceptibility tests

The detection of β-lactamases produced by the AMP^r^ isolates was performed by adding nitrocefin (Oxoid, Hants, England) to the cell biomass, according to the manufacturer instructions. For the positive nitrocefin strains, the susceptibility pattern determination was performed by the disk diffusion method (DDM) on Mueller Hinton agar (Oxoid, Hampshire, England) with antibiotic disks (Oxoid, Hampshire, England) according to the Clinical Laboratory Standards Institute (CLSI), formerly National Centre Clinical Laboratory Standards (NCCLS) [15,16]. Thirteen different antibiotics were used: amoxicillin/clavulanic acid combination (Clavamox) (AMC) 30 μg/10 μg, respectively; ceftazidime (CAZ) 30 μg; cefotaxime (CTX) 30 μg; cefpirome (CPO) 30 μg; aztreonam (ATM) 30 μg; cefoxitin (FOX) 30 μg; imipenem (IPM) 10 μg; meropenem (MEM) 10 μg; chloramphenicol (CHL) 30 μg; tetracycline (TET) 30 μg; gentamicin (GEN) 10 μg; trimethoprim/sulfamethoxazol (Bactrim®; SXT) combination (1:19) and ciprofloxacin (CIP) 5 μg.

In order to observe the influence of clavulanic acid on both CAZ and CTX, these antibiotics were positioned in line, with (AMC) in the middle. The results of the antimicrobial susceptibilities were interpreted according to the CLSI guidelines.

### 2.5. Bacterial identification

The identification of sixty β-lactamase producing isolates randomly selected from VRBG and SS plates was carried out in two steps. Firstly, classical morphological and physiological tests were performed in agreement with the Bergey’s Manual of Determinative Bacteriology [17]. These tests included Gram staining reaction, morphology, motility, and biochemical tests including: fermentative/oxidative metabolism of glucose [18] (fermentation of glucose to acid or to acid and gas), aerobic/anaerobic growth, catalase and oxidase reactions. Subsequently, the isolates identified as belonging to the Enterobacteriaceae family were submitted to additional identification by using the miniaturized Enterosystem 18R galleries (Liofilchem Bacteriology Products, Roseto, Italy), following the manufacturer instructions. Enterosystem 18 R system is based on the metabolism of orthonitrophenol (ONPG), lysine, ornithine, arginine-decarboxylase, phenilalanine-deaminase, citrate, malonate, urease test, H_2_S, and Voges-Proskauer reaction, indol test and in the fermentation of glucose, mannitol, inositol, sorbitol, sucrose, arabinose and raffinose.

### 2.6. β-lactamase genes

The identification of the genes involved in β-lactam resistance was performed, in 21 random selected β-lactamase producing isolates, by a multiplex-PCR approach for three β-lactamase (*bla*) genes (*bla*_TEM_, *bla*_SHV_ and *bla*_CTX-M_) according to Monstein *et al*. [19], except that genomic DNA was extracted by the guanidine thiocyanate method according to Pitcher *et al.* [20].

Strains *E. coli* UB0402407 and *K. pneumoniae* HY0301692, kindly provided by the Clinical Microbiology Department, Linköping University, Sweden, were used as positive controls for *bla*_TEM_ + *bla*_CTX-M_ genes and *bla*_SHV_ gene, respectively.

## 3. Results and Discussion

### 3.1. Enumeration of ampicillin resistant bacteria

**Figure 1.**
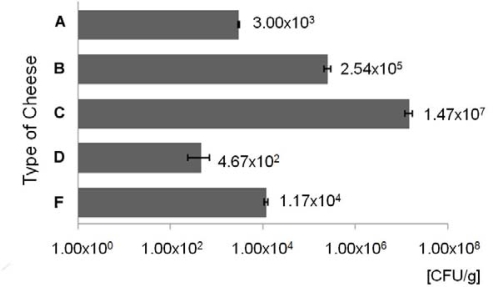
Mean values of ampicillin resistant (AMP^r^) bacteria (CFU/g of cheese) ± the standard deviation (error bars), on VRBG agar medium, after 38 hours at 37 ºC.

The enumeration of ampicillin resistant bacteria was performed on VRBG agar, a medium for the detection and enumeration of bile-tolerant Enterobacteriaceae in dairy products and foods, that is becoming a preferred medium for use in investigations into raw materials, processed foods and plant hygiene. Figure 1 shows the counting data of AMP^r^ bacteria on VRBG plates, relative to five cheeses samples corresponding to five different types of cheese (A, B, C, D and F, Table 1). According to the type of cheese, the numbers of AMP^r^ varied from about 4.7 × 10^2^ CFU/g to 1.5 × 10^7^ CFU/g.

Although Enterobacteriacea are used as a parameter in process hygiene criteria for various food products, current legislation in Europe does not use Enterobacteriaceae as microbiological criteria for cheese made from raw milk. Nevertheless, in cheeses made from pasteurized milk the level of contamination with *E. coli* may not exceed 10^3^ CFU/g [21]. Various genera of Enterobacteriaceae are commonly found in milk and cheese [4], although the highest counts close to 10^7^ CFU/g were generally found during the first days of ripening, decreasing afterwards [22]. In this work, the numbers of AMP^r^ bacteria varied according to the type of cheese (Figure 1). The lowest values were obtained for cheese types A (3.0 × 10^3^ CFU/g) and D (4.7 × 10^2^ CFU/g). The first is a DOP (Denomination of Protected Origin) cheese made from raw ewe’s milk, the second is a cheese made from pasteurized goat’s milk (Table 1). The results from the counts in cheese type A are an example that it is possible to produce good quality cheese with raw milk. The results from cheese type D, made from pasteurized goat’s milk confirm that the thermal processing of the milk is an important factor to decrease bacterial contaminations. Nevertheless, to rely only on pasteurization to solve bacterial contaminations is likely to limit efforts to improve total hygiene in cheese production.

The highest level was obtained for type C cheese made from raw goat’s milk (1.5 × 10^7^ CFU/g). Since the majority (85%) of the isolates identified belonged to the Enterobacteriaceae family, the suggested level of contamination of the cheese from type C, deduced from the presented data, is concerning.

In order to avoid high rates of cheese contamination with these and other potential hazardous bacteria, the existence of good sanitary conditions during milking and cheesemaking is obligatory. This includes herd health management, particularly udder health preventive management, hygiene of milking, promptly and properly refrigeration of the milk after milking and hygiene along the cheese production chain.

### 3.2. Detection of β-lactam-resistant bacteria

The detection of AMP^r^ bacteria in cheeses was performed by using VRBG agar and SS agar. In both media, the growth of gram-positive microorganisms is inhibited due to the presence of either pure bile salts, mixtures of bile salts or dyes. SS agar has been developed for the selection and differentiation of enteric microorganisms, from clinical and nonclinical materials.

A total of 182 AMP^r^ selected isolates recovered from VRBG and from SS plates were screened for the presence of β-lactamases by the nitrocefin test. From the 182 isolates, 172 were found positive (102 from VRBG and 70 from SS plates) indicating the presence of β-lactamases in 94.5% of the AMP^r^ isolates. The three isolates selected from cheese type E (made from pasteurized cow’s milk) were within the 10 nitrocefin negative isolates.

Concerning the suitability of both media to recover β-lactam-resistant bacteria, the results showed that both media fulfilled this objective. From a total of sixty β-lactamase producers submitted to identification, only three (6.7% of the isolates) and five (15% of the isolates) species were recovered only from SS or from VRBG plates, respectively (Table 2).

### 3.3. Bacterial identification

Sixty nitrocefin positive isolates (37 from VRBG and 23 from SS plates) were identified as belonging to the Enterobacteriaceae family (85%) and to the genera *Pseudomonas* (8.3%) and *Aeromonas* (6.7%) (Table 2). Within the Enterobacteriaceae family, 34 isolates (12 from SS and 22 from VRBG plates) were identified as belonging to eleven different species. It was not possible to identify at species level 17 Enterobacteriaceae isolates (28.3%) recovered from both media. The type cheese B, made from raw ewe’s milk (Table 1) was the source of most of the identified isolates. However 13 out of the 20 cheeses analyzed were from this type. Five different enterobacterial species were collected from type cheese F (made from a raw milk mixture from cow, ewe and goat) although a single cheese was analyzed. Regarding the recovery medium, from the eleven identified species, five (*Enterobacter cloacae*, *Proteus mirabilis*, *Citrobacter freundii*, *Enterobacter sakasakii* and *Enterobacter alvei*) and three (*Serratia rubidaea*, *Providencia retgeri* and *Shigella boydii*) species were only collected from VRBG or SS plates, respectively (Table 2). *Pseudomonas* and *Aeromonas* isolates were recovered from both media.

### 3.4. Antimicrobial susceptibility tests

The results from antimicrobial susceptibility determination for the 13 antimicrobial agents and the respective percentage of the resistance phenotypes obtained showed a higher resistance to AMC (51.7%) and FOX (46.5%) and to TET (46.5%) (Table 3).

It is also noticed a high resistance level (38.4 %) to SXT. Nevertheless, antimicrobial agents which seem more effective, thus having higher susceptibility among these bacterial strains are GEN (98.3%), CIP (97.7%), IPM (95.3%) and MEM (90.7%) (Table 3). In this study, a multidrug resistance (MDR) phenotype was defined for isolates that were resistant to two or more structurally unrelated antimicrobial agents. According to this, 31.4% of the different phenotypes detected displayed a MDR phenotype (Table 4). The most prevalent resistant phenotypes have patterns of SXT and/or TET plus AMC and FOX and finally CHL.

The most effective antimicrobial agents against the bacterial strains isolated are GEN (98.3%), CIP (97.7%), IPM (95.3%) and MEM (90.7%). It seems noteworthy the high bacterial susceptibility to CIP found since in Portugal, in contrast to other European countries, this type of antibiotics is one of the most frequently used [23]. The high frequency of resistance to AMC (51.7%) might be due to the their extensive usage in Europe, since penicillins represent the most widely used antibiotic class in all 25 participating countries studied by Ferech *et al*. [24] in the ESAC Project Group. These authors reported a distinct shift from narrow-spectrum penicillins to broad-spectrum penicillins, and specifically their combinations with β-lactamase inhibitors, during the period 1997–2003.

On the other hand, the equally high resistance to FOX (46.5%) a cephamycin, is probably because it has been on the market for a longer period which may have allowed for the development of more resistance than those that are more recently available [25]. The shifting in antibiotic use in Europe is extremely high for this class of antibiotics, suggesting that in many European countries these antibiotics are prescribed inappropriately [25].

The high resistance detected to TET (46.5%) may be related with their extensive use in veterinary, [26–29] including aquacultures. Nevertheless, it could also mean an eventual non efficiency in humans through selection, following the widespread use of these antibiotics [27].

The most prevalent resistance phenotype is AMP + TET (11.0%) followed by the MDR phenotypes AMP + SXT + TET and AMP + AMC + FOX + SXT + TET (each 4.7%). This fact might result from the association of TET with the massive use of Bactrim® (SXT) [30] mainly for the treatment of urinary infections [31]. Other sulfonamides are also described as widely used for both veterinary and aquacultures purposes [27]. Among the MDR phenotypes, resistance to CHL was also present in important phenotypes such as AMP + AMC + FOX + CHL + TET (4.1%) and AMP + AMC + FOX +CHL + TET + SXT (2.3%) or AMP + AMC + FOX + CHL + SXT (2.3%).

The relatively high number (31.4%) of MDR phenotypes found among bacteria isolated from different types of cheese, may contribute to the emergence of a serious public health problem. These finding are in agreement with the results of Fernandes *et al*. [32], that reported the spreading of MDR in hospitals and in the community by isolates of *E. coli* coding for ESBLs. The same effect was also reported in other European countries [32,33].

### 3.5. Molecular genetics of resistance genes

Twenty one randomly selected isolates were tested for the presence of three β-lactamases genes. The expected sizes of these amplicons, according to the control strains used, were 445 bp for the *bla*_TEM_, 593 bp for the *bla*_CTX-M_ and 747 bp for the *bla*_SHV_. The multiplex-PCR analysis showed that, while the *bla*_CTX-M_ and *bla*_SHV_ were absent, 80.9% of the tested isolates harbor a gene for the TEM-type enzyme (*bla*_TEM_).

The presence of *bla*_TEM_ in bacteria isolated from cheese reported in this study, is in agreement with several recent reports showing that TEM β-lactamases are particularly involved in resistance phenotypes presented by bacteria isolated from several agro-industrial environments such as in water reservoirs [34], wastewaters [35], livestock [36–39] and even in transgenic plant fields [40]. The presence of TEM β-lactamases in these bacteria may be explained by its insertion between transposable elements that move rapidly through bacterial populations [41] and may become a major public health concern. However, TEM-β-lactamases alone may not explain all the resistance phenotypes found in the present study. Regarding the results presented in Table 3, they may be explained by several other mechanisms that include the presence of other β-lactamases, alteration of membrane permeability such as due the presence of a porin loss, modification of target, efflux mechanisms. In what concerns to the mechanisms related to other antimicrobial rather than β-lactams the mechanisms that may explain these resistance phenotype are identical [5,6]. In order to clarify this subject, further studies are needed.

## 4. Conclusions

Aiming to investigate the role of the consumption of a RTE type of food (cheese) in the transfer of β-lactams-resistant bacteria to the gastrointestinal (GI) flora of the consumer, twenty cheese samples were analysed. These samples corresponded to six different types of Portuguese ripened cheese, collected from retail market at different geographical regions of Portugal.

The numbers of AMP^r^ bacteria recovered from the cheeses varied from 4.7 × 10^2^ CFU/g to 1.5 × 10^7^ CFU/g. Since 85% of the selected isolates were identified as belonging to the Enterobacteriaceae family, the highest counting values obtained (particularly cheese types B and C) suggest the absence of good sanitary conditions during milking and/or cheesemaking. This fact is of considerable public health concern, particularly because the nitrocefine test indicated the presence of β-lactamases in 94.5 % of the AMP^r^ isolates. The multiplex PCR assay indicated a high prevalence (80.9%) of the *bla*_TEM_ gene among the tested isolates. The antimicrobial susceptibility testing values and the resistance phenotypes found among the isolates may be of clinical relevance if the resistance determinants could be transferred to humans. Transfer of either antibiotic-resistant bacteria to humans or of their antibiotic resistance genes to pathogens, *via* the food chain has already been reported [42–44].

Resistance to antibiotics is a major public-health problem and antibiotic use is being increasingly recognised as the main selective pressure driving this resistance [45]. Prescription of antibiotics in primary care in Europe varied greatly nevertheless, a shift was recently noted in the consumption of antibiotics from the old narrow-spectrum antibiotics to the new broad-spectrum antibiotics. High consuming countries showed higher rates of antibiotic resistance, probably related to the higher consumption in Southern and Eastern Europe than in Northern Europe [45].

To our knowledge, this is the first report in Portugal of the presence of β-lactam resistance bacteria in cheese. The results suggest the importance of good hygienic practices during milking and cheesemaking, particularly when the cheese is made from raw milk. In the genome of the carrier bacteria, the localization of the β-lactamase genes needs to be elucidated in order to predict their possibility of transfer to the consumer GI flora.

## Figures and Tables

**Table 1.** Characterization of the cheeses analyzed with respect to the origin and thermal processing of the milk.

Type of cheese	**Milk**		Number of cheeses
	Origin	Thermal processing	
A*	ewe	no	2
B	ewe	no	13
C	goat	no	2
D	goat	pasteurization	1
E	cow	pasteurization	1
F	mixture (cow, ewe and goat)	no	1

* DOP (Denomination of Protected Origin).

**Table 2.** Identification of 60 AMP^r^ isolates positive for the nitrocefin test, and respective type of cheese and recovery medium.

Family/Genus	Species	Number of isolates	%	Type of cheese	Recovery medium
Enterobacteriaceae	NI	17	28.3	A, B, C, D	VRBG, SS
	*Proteus vulgaris*	9	15.0	B, C, F	VRBG, SS
	*Escherichia coli*	6	10.0	B, D, F	VRBG, SS
	*Morganella morganii*	6	10	B, C, F	VRBG, SS
	*Enterobacter cloacae*	4	6.7	A, B, F	VRBG
	*Proteus mirabilis*	2	3.3	B, F	VRBG
	*Serratia rubidaea*	2	3.3	A	SS
	*Citrobacter freundii*	1	1.7	B	VRBG
	*Enterobacter sakazakii*	1	1.7	B	VRBG
	*Enterobacter alvei*	1	1.7	B	VRBG
	*Providencia retgeri*	1	1.7	B	SS
	*Shigella boydii*	1	1.7	D	SS
*Pseudomonas* spp.	NI	5	8.3	B, C	VRBG, SS
*Aeromonas* spp.	NI	4	6.7	B, C, D	VRBG, SS
All		60	100	A, B, C, D, F	VRBG, SS

Legend: Medium VRBG – Violet Red Bile Glucose; Medium SS – *Salmonella/Shigella*. NI – Not identified.

**Table 3.** Minimal inhibitory concentrations (MIC) ^a)^ for the antimicrobial agents tested and percentage of the resistance or susceptible phenotype obtained among the isolated AMP^r^/nitrocefin positive strains (*n* = 172).

		**Susceptible**				**Resistant**			

	Antimicrobial agent	Φ	MIC ^a)^		%	Φ	MIC ^a)^	n	%

		(mm)	(μg/mL)	*n*	(*n* = 172)	(mm)	(μg/mL)		(*n* = 172)
**β - lactams**	AMC (30:10 μg)	≥ 18	≤ 8/4	67	39.0	≤ 13	≥ 32/16	89	51.7
	FOX (30 μg)	≥ 18	≤ 8	70	40.7	≤ 14	≥ 32	80	46.5
	CTX (30 μg)	≥ 23	≤ 8	115	66.9	≤ 14	≥ 32	23	13.4
	CPO (30 μg)	≥ 18	≤ 8	150	87.2	≤ 14	≥ 32	10	5.8
	ATM (30 μg)	≥ 22	≤ 8	142	82.6	≤ 15	≥ 32	10	5.8
	CAZ (30 μg)	≥ 18	≤ 8	158	91.9	≤ 14	≥ 32	9	5.2
	MEM (10 μg)	≥ 16	≤ 4	156	90.7	≤ 13	≥ 16	9	5.2
	IPM (10 μg)	≥ 16	≤ 4	164	95.3	≤ 13	≥ 16	2	1.2

**Non β – lactams**	TET (30 μg)	≥ 19	≤ 4	56	32.6	≤ 14	≥ 16	80	46.5
	SXT (1:19 μg)	≥ 16	≤ 2/38	103	59.9	≤ 10	≥ 8/152	66	38.4
	CHL (30 μg)	≥ 18	≤ 8	108	62.8	≤ 12	≥ 32	35	20.3
	GEN (10 μg)	≥ 15	≤ 4	169	98.3	≤ 12	≥ 8	3	1.7
	CIP (5 μg)	≥ 21	≤ 1	168	97.7	≤ 15	≥ 4	0	0.0

a) MIC values were determined after conversion of the respective diameters obtained from the disc diffusion antimicrobial susceptibility testing from CLSI standards [15,16].

**Table 4.** Prevalence of resistance phenotypes to major antimicrobial classes among 172 nitocefin positive isolates.

	Resistance Phenotype	Number of isolates	%
**Multidrug resistant (MDR) phenotypes**	AMP, SXT, TET	8	4.65
	AMP, AMC, FOX, SXT, TET	8	4.65
	AMP, AMC, FOX, CHL, TET	7	4.07
	AMP, AMC, FOX, CHL, SXT, TET	4	2.33
	AMP, AMC, FOX, CHL, SXT	4	2.33
	AMP, AMC, CHL, TET	2	1.16
	AMP, CHL, SXT, TET	2	1.16
	AMP, AMC, SXT, TET	2	1.16
	AMP, AMC, CHL, SXT, TET	2	1.16
	AMP, AMC, FOX, CEPH3, CPO, CHL, SXT, TET	2	1.16
	AMP, CHL, TET	2	1.16
	AMP, FOX, CHL, TET	2	1.16
	Others with only one representative	9	5,22

**Sub-total**		54	31.40

**Non-multidrug resistant (MDR) phenotypes**	AMP	38	22.09
	AMP, TET	19	11.05
	AMP, AMC, FOX, SXT	8	4.65
	AMP, AMC, FOX, TET	7	4.07
	AMP, AMC, FOX	6	3.49
	AMP, AMC, TET	5	2.91
	AMP, SXT	5	2.91
	AMP, AMC	4	2.33
	AMP, AMC, FOX, CEPH3, ATM	4	2.33
	AMP, AMC, FOX, CEPH3, CPO, ATM	4	2.33
	AMP, FOX, SXT	2	1.16
	AMP, AMC, FOX, CEPH3, CARB, SXT	2	1.16
	AMP, AMC, FOX, CEPH3, SXT	2	1.16
	AMP, AMC, FOX, CEPH3, CPO, CARB, SXT	2	1.16
	AMP, AMC, FOX, SXT	2	1.16
	Others with only one representative	8	4.64

**Sub-Total**		118	68.60

**Total**		172	100
